# Efficient Sampling of Knotting-Unknotting Pathways for Semiflexible Gaussian Chains

**DOI:** 10.3390/polym9060196

**Published:** 2017-05-29

**Authors:** Cristian Micheletti, Henri Orland

**Affiliations:** 1 International School for Advanced Studies (SISSA), Physics Area via Bonomea 265, I-34136 Trieste, Italy; 2 Institut de Physique Théorique, CEA, CNRS, UMR3681, F-91191 Gif-sur-Yvette, France; 3 Beijing Computational Science Research Center, No.10 East Xibeiwang Road, 100193 Beijing, China

**Keywords:** knots, Langevin bridges, reconnection paths

## Abstract

We propose a stochastic method to generate exactly the overdamped Langevin dynamics of semi-flexible Gaussian chains, conditioned to evolve between given initial and final conformations in a preassigned time. The initial and final conformations have no restrictions, and hence can be in any knotted state. Our method allows the generation of statistically independent paths in a computationally efficient manner. We show that these conditioned paths can be exactly generated by a set of local stochastic differential equations. The method is used to analyze the transition routes between various knots in crossable filamentous structures, thus mimicking topological reconnections occurring in soft matter systems or those introduced in DNA by topoisomerase enzymes. We find that the average number of crossings, writhe and unknotting number are not necessarily monotonic in time and that more complex topologies than the initial and final ones can be visited along the route.

## 1. Introduction

Filamentous systems are typically strongly affected by topological constraints in their conformational, mechanical and dynamical properties. This is especially evident for self-avoiding ring polymers, which are trapped in a specific knotted state that cannot be altered in the course of their free dynamical evolution. For these reasons, much interest has been spurred recently by the dramatic topological changes observed in crossable filamentous structures that can appear in dissipative systems. Notable instances include entangled optical beams [1,2], vortex lines in fluids [3], magnetic field lines in a plasma [4] and defect lines in liquid crystals [5,6,7,8,9,10]. By contrast to polymers, strand crossings can occur when these filaments collide (subject to specific local conservation rules [11,12,13]), thus creating the conditions for dynamical changes in topology.

Significant efforts are being made to map out the possible reconnection pathways and establish their recurrence across various dissipative systems [14,15]. A key related question is whether the observed modes of topological changes are any different from those sustained by semi-flexible phantom rings. These systems, in fact, serve as terms of reference to understand the action of topoisomerase enzymes. These are enzymes that can progressively simplify the global knotted topology of DNA rings by fostering suitable local strand passages. Many efforts are accordingly made to understand which local selection criteria for strand passage would have the same disentangling effects on knotted phantom rings [16,17,18,19,20,21].

Advancements along these lines depend, at least in part, on the possibility to generate computationally, or predict theoretically, physically-viable trajectories connecting two conformations with preassigned topology. This task is, in general, very challenging because spontaneous dynamical evolutions from a given initial state are unlikely to end up in a preassigned target one within a finite computational time, especially when significant free energy barriers are present along the route. Such difficulties are usually tackled by accelerating the dynamics using path sampling methods [22,23,24,25,26,27,28,29,30] or with steered molecular dynamics techniques based on suitable external, and possibly time-dependent forces [31,32,33,34,35,36]. These schemes have proved essential for profiling free energy landscapes and establishing the salient steps along transition pathways. At the same time, they usually do not leave good control over the probabilistic weight of the trajectories, and hence on their representative significance.

Here we present a novel theoretical, and computationally efficient scheme based on Langevin bridges [37,38] that allows one to connect two states with preassigned geometry by means of unbiased and physically-viable trajectories. With this strategy, that is entirely general, we are able to study in great detail the canonically-relevant transitions pathways of semi-flexible rings between two assigned conformations of any topology. We show that these canonical transition pathways are often not minimal, meaning that more complex topologies than the initial and final ones can be visited along the route. This exposes an unsuspectedly rich phenomenology of topological rearrangements that could be explored and verified in future experiments on entangled soft matter systems.

## 2. Methods

### 2.1. The Conditioned Langevin Equation

For the sake of simplicity, we start by illustrating the method on a one-dimensional system, following closely the presentation given in ref. [37]. We assume that the system is driven by a force F(x,t) and is subject to stochastic dynamics in the form of an overdamped Langevin equation:

(1) $$\frac{dx}{dt}=\frac{1}{\gamma }F\left(x\left(t\right),t\right)+\eta \left(t\right)$$

where x(t) is the position of the particle at time *t* which experiences the force F(x,t). The friction coefficient γ is related to the particle diffusion coefficient *D* through the Einstein relation $D=k_{B}T/\gamma$, where $k_{B}$ is the Boltzmann constant and *T* the temperature of the thermostat. Finally, η(t) is a Gaussian white noise with moments given by 〈η(t)〉=0 and $\langle \eta \left(t\right)\eta \left(t^{\prime }\right)\rangle =2\;D\;\delta \left(t-t^{\prime }\right)$.

One can show (see refs. [37], and Supplementary Materials) that the Langevin trajectories starting at x=0 at time t=0 and conditioned to end at $x_{f}$ at time $t_{f}$, can be generated by a Langevin equation with an additional potential force

(2) $$\frac{dx}{dt}=\frac{1}{\gamma }F+2D\frac{\partial \ln Q}{\partial x}+\eta \left(t\right)$$

where

(3) $$Q\left(x,t\right)=P\left(x_{f},t_{f}|x,t\right)$$

and $P(x_{f},t_{f}|x,t)$ denotes the probability to find the particle at $x_{f}$ at time $t_{f}$, given that it was at *x* at time *t*.

This equation generates Brownian paths, starting at $\left(x_{0},0\right)$ conditioned to end at $\left(x_{f},t_{f}\right)$, with unbiased statistics. It is the additional term $2D\frac{\partial \ln Q}{\partial x}$ in the conditioned Langevin equation that guarantees that the trajectories starting at $\left(x_{0},0\right)$ will end at $\left(x_{f},t_{f}\right)$ and are statistically unbiased.

Equation (2) is straightforwardly generalized to systems with many degrees of freedom. Specifically, for systems comprising *N* particles interacting via a potential *U* and subject to an external force $F_{n}$ acting on particle *n*, the evolution of the position vector $r_{n}$ of the *n*th particle, is given by:

(4) $$\frac{dr_{n}}{dt}=-\frac{1}{\gamma }\nabla_{r_{n}}U+\frac{1}{\gamma }F_{n}\left(t\right)+2D\nabla_{r_{n}}\ln Q\left(\left\{r_{n}\right\},t\right)+\eta_{n}\left(t\right)$$

where $Q\left(\left\{r_{n}\right\},t\right)=P\left(\left\{r_{n}^{\left(f\right)}\right\},t_{f}|\left\{r_{n}\right\},t\right)$ and the Gaussian noise $\eta_{n}\left(t\right)$ satisfies

(5) $$\begin{array}{cc} \langle \eta_{n}^{\left(\alpha \right)}\left(t\right)\rangle =0,\;\;\; & \langle \eta_{n}^{\left(\alpha \right)}\left(t\right)\eta_{n^{\prime }}^{\left(\alpha^{\prime }\right)}\left(t^{\prime }\right)\rangle =\frac{2\;k_{B}T}{\gamma }\delta_{nn^{\prime }}\delta_{\alpha \alpha^{\prime }}\delta \left(t-t^{\prime }\right) \end{array}$$

where α labels the Cartesian coordinates x,y,z.

### 2.2. Polymer Chain

We now specialize Equation (4) to the case of ring polymers that freely evolve under the action of the following inter-monomer potential, *U*

$$\frac{U}{k_{B}T}=\sum_{n=1}^{N}\left[\frac{3}{2a^{2}}{\left(r_{n+1}-r_{n}\right)}^{2}+\frac{K}{2}{\left(r_{n+1}-2r_{n}+r_{n-1}\right)}^{2}\right]$$

where $r_{N}=r_{0}$ and $r_{N+1}=r_{1}$, since the chain is a ring.

The first term is the elasticity of the polymer chain, whereas the second represents its bending rigidity. This expression for the bending rigidity is approximate, since the monomer length is not fixed in this model. However, this is a standard mean-field type model to represent semi-flexible polymers. We further assume no external force, $F_{n}\left(t\right)=0$.

To model chains with preassigned root-mean-square bond length, *b*, and persistence length, $l_{P}$, the bare parameters *a* and *K* must be set by solving the following equations:

(6) $$\begin{array}{c} l_{P}=\sqrt{\frac{Ka^{2}}{3}}\;\\ b^{2}=\frac{1}{N}\langle \sum_{n=1}^{N}{\left(r_{n+1}-r_{n}\right)}^{2}\rangle \;\\ \;\;\;\;=\frac{a^{2}}{N}\sum_{p=0}^{N-1}{\left[1+\frac{2Ka^{2}}{3}\left(1-\cos\omega_{p}\right)\right]}^{-1} \end{array}$$

where $\omega_{p}=\frac{2\pi }{N}p$. For large *K* and sufficiently long chains, Equation (6) yields the expected linear dependence of the persistence length on the chain bending rigidity (see Supplementary Materials).

For the considered polymer case, the Langevin bridge equation of Equation (4) is best expressed in Fourier space:

(7) $$\frac{d{\tilde{\rho }}_{p}}{dt}=-\Omega_{p}{\tilde{\rho }}_{p}+\frac{D}{N}\nabla_{{\tilde{\rho }}_{p}}\log Q+{\tilde{\eta }}_{p}$$

where

(8) $$\begin{array}{ccc} {\tilde{\rho }}_{p} & = & \frac{2}{N}\sum_{n=1}^{N}\cos\left(\omega_{p}n\right)\;r_{n} \end{array}$$

(9) $$\begin{array}{ccc} \Omega_{p} & = & \left(3/a^{2}\right)\;\left(1-\cos\omega_{p}\right)+2K{\left(1-\cos\omega_{p}\right)}^{2} \end{array}$$

and ${\tilde{\eta }}_{p}$ are the Fourier series of $\eta_{n}\left(t\right)$ and are thus Gaussian white noises, defined by their moments

(10) $$\begin{array}{ccc} \langle {\tilde{\eta }}_{p}^{\alpha }\left(t\right)\rangle & = & 0 \end{array}$$

(11) $$\begin{array}{ccc} \langle {\tilde{\eta }}_{0}^{\left(\alpha \right)}\left(t\right){\tilde{\eta }}_{p}^{\left(\alpha^{\prime }\right)}\left(t^{\prime }\right)\rangle & = & \frac{2D}{N}\delta_{p0}\delta_{\alpha \alpha^{\prime }}\delta \left(t-t^{\prime }\right) \end{array}$$

(12) $$\begin{array}{ccc} \langle {\tilde{\eta }}_{p}^{\left(\alpha \right)}\left(t\right){\tilde{\eta }}_{p^{\prime }}^{\left(\alpha^{\prime }\right)}\left(t^{\prime }\right)\rangle & = & \frac{D}{N}\delta_{pp^{\prime }}\delta_{\alpha \alpha^{\prime }}\delta \left(t-t^{\prime }\right)\;. \end{array}$$

The Green’s function $Q({\tilde{\rho }}_{p},t)$ can be computed exactly by solving the Langevin equation in Fourier space. The calculation is straightforward, see Supplementary Materials, and yields the following bridge equation

(13) $$\frac{d{\tilde{\rho }}_{0}}{dt}=\frac{{\tilde{\rho }}_{0}^{\left(f\right)}-{\tilde{\rho }}_{0}\left(t\right)}{t_{f}-t}+{\tilde{\eta }}_{0}\left(t\right)$$

(14) $$\begin{array}{c} \frac{d{\tilde{\rho }}_{p}}{dt}=-\Omega_{p}{\tilde{\rho }}_{p}\left(t\right)\;\\ \;\;\;\;\;\;+\frac{\Omega_{p}}{\sinh[\Omega_{p}\left(t_{f}-t\right)]}\left({\tilde{\rho }}_{p}^{\left(f\right)}-{\tilde{\rho }}_{p}\left(t\right)e^{-\Omega_{p}\left(t_{f}-t\right)}\right)\;\\ \;\;\;\;\;\;+{\tilde{\eta }}_{p}\left(t\right) \end{array}$$

where $\Omega_{p}=\left(3/a^{2}\right)\;\left(1-\cos\omega_{p}\right)+2K{\left(1-\cos\omega_{p}\right)}^{2}$ and ${\tilde{\rho }}_{p}^{\left(f\right)}$ denotes the final configuration of the chain in Fourier components. These equations can be discretized and solved numerically, from an initial configuration ${\tilde{\rho }}_{p}^{\left(0\right)}$ to a final one ${\tilde{\rho }}_{p}^{\left(f\right)}$.

We point out that the time-reversed trajectory is a legitimate solution of the bridge equations starting from $\left\{r^{\left(f\right)}\right\}$ at time t=0 and ending in $\left\{r^{\left(0\right)}\right\}$ at time $t=t_{f}$. Also note that within this model, the contour length of the chain is not conserved during the time evolution. For representation purposes, it is possible to rescale the contour length to its initial value at any given time *t* when inverting back from Fourier to real space representation.

### 2.3. Circular Permutations

In the bridge Equations (13) and (14), monomers in the initial and final states are in one-to-one correspondence. To study the evolution between two ring shapes in the absence of external forces, one should allow the initial configuration $\left\{r_{1}^{\left(0\right)},\ldots ,r_{n}^{\left(0\right)}\right\}$ to go to any circular permutation of the final configuration, i.e. $\left\{r_{1+n_{0}}^{\left(f\right)},\ldots ,r_{N+n_{0}}^{\left(f\right)}\right\}$, for any $n_{0}=0,\ldots ,N-1$, where we assume periodic conditions since the chain is a ring $r_{n+N}=r_{n}$. This requires substituting the single final state with a combination of its circular permutations. It has been shown [37] that if the final state is a combination of several states, the function *Q* should be modified as

(15) $$Q\left(\left\{r_{n}\right\},t\right)=\sum_{n_{0}=0}^{N-1}P\left(\left\{r_{n+n_{0}}^{\left(f\right)}\right\},t_{f}|\left\{r_{n}\right\},t\right)$$

so that in absence of an external force, the bridge equations become

(16) $$\frac{d{\tilde{\rho }}_{0}}{dt}=\frac{{\tilde{\rho }}_{0}^{\left(f\right)}-{\tilde{\rho }}_{0}\left(t\right)}{t_{f}-t}+{\tilde{\eta }}_{0}\left(t\right)$$

(17) $$\begin{array}{c} \frac{d{\tilde{\rho }}_{p}}{dt}=-\Omega_{p}{\tilde{\rho }}_{p}\left(t\right)+\frac{\Omega_{p}}{\sinh[\Omega_{p}\left(t_{f}-t\right)]}\frac{1}{Q}\sum_{n_{0}=1}^{N-1}\left({\tilde{\rho }}_{p}^{\left(n_{0}\right)}-\right.\;\\ \;\;\;\;\;\;\;\left.{\tilde{\rho }}_{p}\left(t\right)e^{-\Omega_{p}\left(t_{f}-t\right)}\right)P_{1}^{\left(n_{0}\right)}+{\tilde{\eta }}_{p}\left(t\right)\;. \end{array}$$

where

(18) $${\tilde{\rho }}_{p}^{\left(n_{0}\right)}=\frac{2}{N}\sum_{n=1}^{N}\cos\left(\omega_{p}n\right)\;r_{n+n_{0}}^{\left(f\right)}$$

and

$$P_{1}^{\left(n_{0}\right)}=\exp\left(-\frac{N}{D}\sum_{p=1}^{N-1}\Omega_{p}\frac{{\left({\tilde{\rho }}_{p}^{\left(n_{0}\right)}-{\tilde{\rho }}_{p}\left(t\right)e^{-\Omega_{p}\left(t_{f}-t\right)}\right)}^{2}}{1-e^{-2\Omega_{p}\left(t_{f}-t\right)}}\right)$$

Similarly to the case without circular permutation, these equations are easily solved by discretization. The numerical complexity is increased due to the summation over circular permutations in Equation (17).

## 3. Results and Discussion

We used the Langevin bridging scheme to connect various pairs of ring polymer conformations tied in different knot types. The initial and final structures were picked from an equilibrated distribution (generated with a Monte Carlo scheme) of self-avoiding semi-flexible rings. These were modelled as a succession of N=240 cylinders with diameter σ=b/4, where *b* is the length of the cylinder axis, and nominal Kuhn length equal to 10b. For integrating the dynamics, and presenting the results, we took *b* as the unit of length, and $D^{2}/b$ as the unit of time. In these units, the dynamics was integrated with a time step equal to $10^{-4}$ and for a total timespan equal to 2.

The excluded volume interactions between the cylinders were then switched off during the Langevin bridging dynamics to allow for topology-unrestricted interconversions. By doing so we model the interconversions observed for defect lines in liquid crystals or vortex lines in fluids in the simplest possible manner. In the mentioned systems, in fact, self-crossings events have an energy cost or are subject to local conservation laws. In this first study, we neglect such interactions to keep the model amenable to extensive theoretical treatment and hence clarify the physically-viable reconnections routes in the simplest and most general setup.

We first discuss the transition from an unknotted conformation to a knotted one, and specifically to a left-handed $5_{1}$ knot. This topology belongs to the family of torus knots, which are drawable without self-intersections on the surface of a torus [39]. We chose it as a first example, because it is the simplest knot type with unknotting number equal to 2. This means that, even in the most favorable conditions, the transition from the trivial to the $5_{1}$ topology cannot occur via a single strand passage, but at least two are needed. This ought to yield interesting knotting pathways.

**Figure 1:**
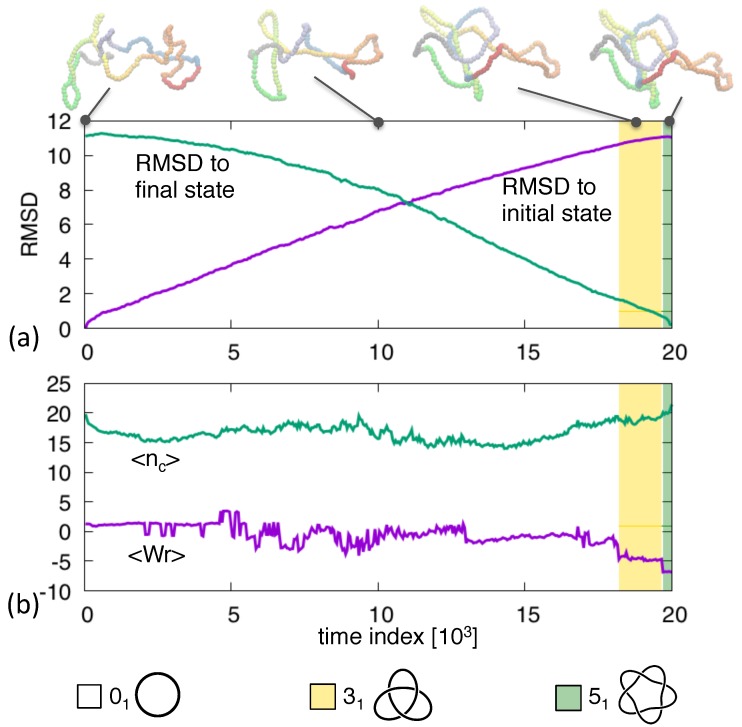
Transition pathway between an unknotted ring and a left-handed $5_{1}$ knotted ring. The root-mean-square distance (RMSD) to the initial and final structures at various stages of the trajectory are shown in panel (**a**). Instantaneous configurations at selected times are highlighted. The average crossing number and writhe are shown in panel (**b**). The overlayed colored background indicates the non-trivial topological states, see legend.

An overview of the typical transition pathway between these two conformations is given in Figure 1, where the initial unknotted and final $5_{1}$-knotted conformations are represented along with intermediate snapshots.

The pathway progresses steadily between these states. This is clarified by the time evolution of the root-mean-square distance (RMSD) from the start and end conformations, which progresses steadily and without lag phases, see panel (a). Panel (b), instead, profiles other topology-related metric properties, such as the average crossing number, $\langle n_{c}\rangle$, and the average writhe, 〈Wr〉. We recall that both quantities are obtained by considering several (1000 in our case) two-dimensional projections of the oriented conformation and averaging over them a weighted sum of the projected crossings. For the crossing number each crossing carries the same +1 weight, while for the writhe the weight is either +1 or -1 depending on the handedness (right-hand rule) of the pair of crossings strands [39]. The time evolution of the two quantities is noticeably noisier than the RMSD profile and its overall trend does not show a steady progression from initial to final state. The negative values of 〈Wr〉 in the final stages of the trajectories are consistent with the left-handedness of the target $5_{1}$ knot.

These properties clarify a posteriori that the imposed duration of the transition pathway is adequate: it is not so long that the conformations diffuses randomly away from the initial state before pointing towards the final state, and yet it is not so short that stochastic fluctuations are suppressed.

The associated discontinuous evolution of the topological, knotted state is highlighted by overlaid colored bands in Figure 1. For most of the evolution, the conformation is locked in the unknotted state and becomes non trivial only in the last ∼20% of the trajectory. In this latter part, the $5_{1}$ state is reached via a different, intermediate topology, namely a $3_{1}$ knot. This is consistent with previous considerations on the unknotting number because the $3_{1}$ or trefoil knot has unknotting number equal to 1 and, being the simplest knot type, can optimally bridge between the $0_{1}$ and $5_{1}$ end states. In more general terms, knot transitions can occur only within pairs of knots at strand passage distance equal to 1 [40].

**Figure 2:**
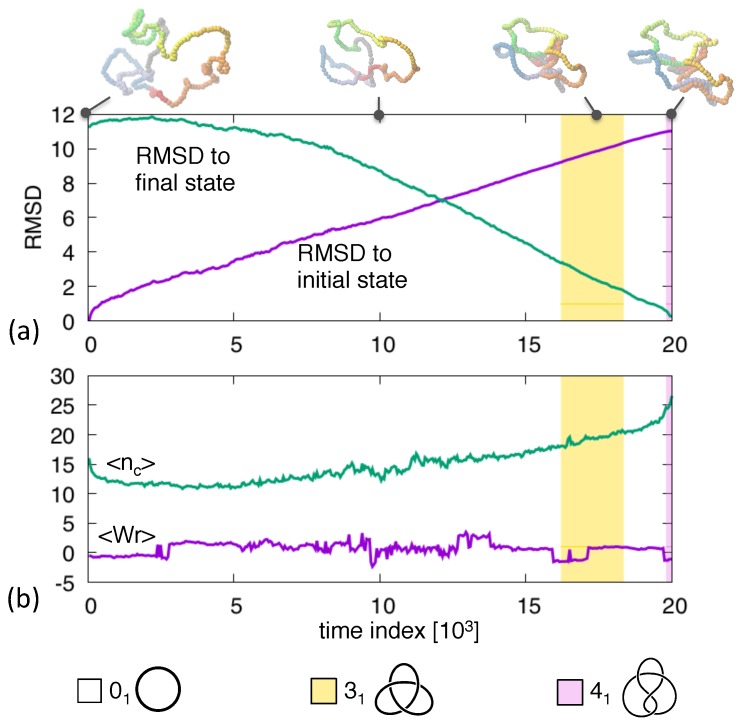
Transition pathway between an unknotted ring and a $4_{1}$ knotted ring. The shown observables are the same as in Figure 1.

This clear and intuitive progression of topological complexity is not always observed. For instance, in Figure 2 one notes that the pathway connecting the shown $0_{1}$ and $4_{1}$ states switches repeatedly between unknotted and $3_{1}$ topologies before reaching the the target figure-of-eight one. The intermittent occupation of trefoil knots is a robust feature of $0_{1}↔4_{1}$ routes. In fact, though direct $0_{1}↔4_{1}$ are clearly possible [40,41] the mediation through $3_{1}$ knots is observed in 10 out of 32 trajectories connecting various combinations of equilibrated initial and final states with $0_{1}$ and $4_{1}$ topologies.

The observed properties of this prototypical interconversion illustrate well the insight that can be gained from Langevin bridging schemes and that would not be obtainable by alternative means.

For instance, allowing the system to evolve freely from the initial state would be ineffective to reach the target topology unless it is highly represented in the canonical ensemble. *A fortiori* the chance that the specific target geometry is reached would practically be always negligible.

Master equation approaches based on transition rates between knot types (observed in a large number of free stochastic evolutions [41]) would be inapplicable too. Transition matrices can correctly capture that the unknot can be directly interconverted to topologies with unknotting number equal to 1 ($3_{1}$, $4_{1}$, $5_{2}$, $6_{1}$, etc.), but the predicted Markov succession of discrete topologies, and their lifetimes, connecting $0_{1}$ to $4_{1}$ states would have no bearings on the actual conformational evolution of ring polymers. The transition matrix approach, therefore, can elegantly recapitulate the equilibrium knotting statistics in terms of Markovian transition between topologies, giving valuable insight into the interplay of geometry and topology. However, generating viable canonical pathways connecting actual states would be beyond its scope. This is were the specificity of the proposed Langevin bridging scheme lies.

From this standpoint, particularly interesting are the transitions between equilibrated rings with different conformations but same topology. From such pathways one can understand whether iso-topological transitions occur via pathways that maintain the same knotted state at all times. Our analysis of 270 trajectories using the same knot type (of up to 5 crossings) for both end states, indicates that the trajectories are not constrained within a single topology.

**Figure 3:**
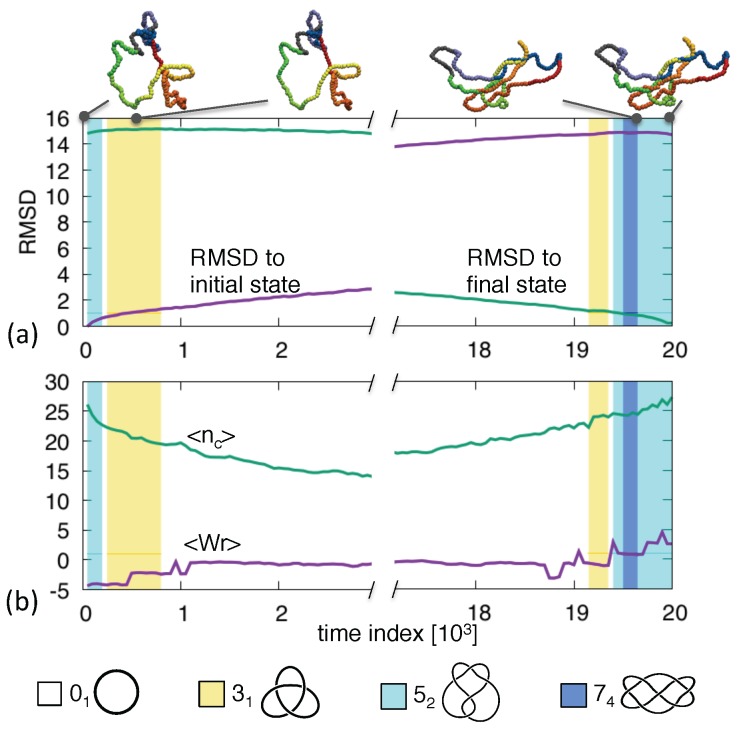
Transition pathway between two $5_{2}$ knotted ring. The shown observables are the same as in Figure 1.

Figure 3 shows one such trajectory with end states tied in a $5_{2}$ knot (same chirality). The bridging pathway clearly populates knots that are simpler ($3_{1}$) and more complex ($7_{4}$) than the initial and final topologies. The presence of $7_{4}$ knots on the route is particularly noteworthy because—unlike the $5_{2}$ one—it has unknotting number equal to 2. This means that the system evolves through states that are definitely more entangled than the initial one and these, in turn, are further simplified before the target state can be reached. This larger-than-expected intermediate complexity is frequent. In our set of 270 trajectories with end states having the same topology of up to 5 crossings, we observed that 6% of the canonical trajectories went through states with 6 or more crossings. The most recurrent type of such knots were $6_{1}$, $6_{2}$ and the aforementioned $7_{4}$.

## 4. Concluding Remarks

In this study, we showed that Langevin bridging schemes provide an effective and elegant solution to the challenging problem of generating viable canonical trajectories between two assigned knotted configurations. The duration of the trajectories can also be specified a priori, thus allowing full control over the system and the simulation cost.

The method allowed us to explore transition pathways between various combination of ring conformations of all topologies up to 5 crossings. We established that such pathways often, though not always, involve intermediate topological states that are more complex than either of the connected states.

We envisage that extensions of this scheme ought to be particularly useful to study the reconnection events that take place spontaneously in dissipative systems of fluctuating crossable filaments and flux tubes. This would require a multicanonical generalization of the approach to deal with a time-dependent number of components.

## Supplementary Materials

The following are available online at www.mdpi.com/2073-4360/9/6/196/s1: Detailed derivation of the bridge equations.
