# Supplementary material for: Efficient Sampling of Knotting-Unknotting Pathways for Semiflexible Gaussian Chains
Source: Polymers (Basel). 2017 May 29;9(6):196. doi: 10.3390/polym9060196 (PMC6432015; doi:10.3390/polym9060196)
Supplement: Supplementary file 1 [file polymers-09-00196-s001.pdf]

Supplemental Material
Efficient sampling of knotting-unknotting pathways for semiflexible Gaussian chains

Cristian Micheletti[1] and Henri Orland[2]
[1] SISSA, via Bonomea 265, I-34136, Trieste, Italy
[2] Institut de Physique Theorique,
CEA, IPhT CNRS, URA2306,
F-91191 Gif-sur-Yvette, France
and
Beijing Computational Science Research Center,
Building 9, East Zone, ZPark II,
No.10 East Xibeiwang Road,
Haidian District, Beijing 100193, China

(Dated: May 17, 2017)

Derivation of the bridge equation

We assume that the system is driven by a force $F(x,t)$ and is subject to stochastic dynamics in the form of an overdamped Langevin equation.

For the sake of simplicity, we illustrate the method on a one-dimensional system, the generalization to higher dimensions or larger number of degrees of freedom being straightforward. We follow closely the presentation given in Ref. [1].

The overdamped Langevin equation reads

$$\frac{dx}{dt} = \frac{1}{\gamma} F(x(t),t) + \eta(t) \tag{1}$$

where $x(t)$ is the position of the particle at time t, driven by the force $F(x,t)$, γ is the friction coefficient, related to the diffusion coefficient D through the Einstein relation $D = k_B T/\gamma$, where k_B is the Boltzmann constant and T the temperature of the heat bath. In addition, $\eta(t)$ is a Gaussian white noise with moments given by

$$\langle \eta(t) \rangle = 0 \tag{2}$$

$$\langle \eta(t)\eta(t') \rangle = \frac{2k_B T}{\gamma}\, \delta(t-t') \tag{3}$$

The probability distribution $P(x,t)$ for the particle to be at point x at time t satisfies a Fokker-Planck equation [2, 3],

$$\frac{\partial P}{\partial t} = D \frac{\partial}{\partial x}\left(\frac{\partial P}{\partial x} - \beta F P \right) \tag{4}$$

where $\beta = 1/k_B T$ is the inverse temperature. This equation is to be supplemented by the initial condition $P(x,0) = \delta(x-x_0)$, where the particle is assumed to be at x_0 at time $t=0$. To emphasize this initial condition, we will often use the notation $P(x,t) = P(x,t|x_0,0)$.

We now study the probability over all paths starting at x_0 at time 0 and conditioned to end at a given point x_f at time t_f, to find the particle at point x at time $t \in [0, t_f]$. This probability can be written as

$$\mathcal{P}(x,t) = \frac{1}{P(x_f,t_f|x_0,0)} Q(x,t) P(x,t)$$

where we use the notation

$$P(x,t) = P(x,t|x_0,0)$$

$$Q(x,t) = P(x_f,t_f|x,t)$$

Indeed, the probability for a path starting from $(x_0,0)$ and ending at (x_f,t_f) to go through x at time t is the product of the probability $P(x,t|x_0,0)$ to start at $(x_0,0)$ and to end at (x,t) by the probability $P(x_f,t_f|x,t)$ to start at (x,t) and to end at (x_f,t_f).

The equation satisfied by P is the Fokker-Planck equation mentioned above (4), whereas that for Q is the so-called reverse or adjoint Fokker-Planck equation [2, 3] given by

$$\frac{\partial Q}{\partial t} = -D \frac{\partial^2 Q}{\partial x^2} - D\beta F \frac{\partial Q}{\partial x} \tag{5}$$

It can be easily checked that the conditional probability $\mathcal{P}(x,t)$ satisfies a new Fokker-Planck equation

$$\frac{\partial \mathcal{P}}{\partial t} = D \frac{\partial}{\partial x}\left(\frac{\partial \mathcal{P}}{\partial x} - \left(\beta F + 2 \frac{\partial \ln Q}{\partial x} \right) \mathcal{P} \right)$$

Comparing this equation with the initial Fokker-Planck (4) and Langevin (1) equations, one sees that it can be obtained from a Langevin equation with an additional potential force

$$\frac{dx}{dt} = \frac{1}{\gamma}F + 2D\frac{\partial \ln Q}{\partial x} + \eta(t) \quad (6)$$

This equation has been previously obtained using the Doob transform [4, 5] in the probability literature and provides a simple recipe to construct a *generalized bridge*. It generates Brownian paths, starting at $(x_0, 0)$ conditioned to end at (x_f, t_f), with unbiased statistics. It is the additional term $2D\frac{\partial \ln Q}{\partial x}$ in the Langevin equation that guarantees that the trajectories starting at $(x_0, 0)$ and ending at (x_f, t_f) are statistically unbiased. This equation can be easily generalized to any number of degrees of freedom.

Equation (6) is straightforwardly generalized to systems with many degrees of freedom. For systems comprising N particles interacting via a potential U and subject to an external force $\mathbf{F}_n$ acting on particle n, the evolution of the position vector $\mathbf{r}_n$ of the nth particle, is given by:

$$\frac{d\mathbf{r}_n}{dt} = -\frac{1}{\gamma}\boldsymbol{\nabla}_{\mathbf{r}_n}U + \frac{1}{\gamma}\mathbf{F}_n(t) + 2D\boldsymbol{\nabla}_{\mathbf{r}_n}\ln Q + \boldsymbol{\eta}_n(t) \quad (7)$$

where $Q(\{\mathbf{r}_n\}, t) = P\left(\{\mathbf{r}_n^{(f)}\}, t_f \mid \{\mathbf{r}_n\}, t\right)$, $\{\mathbf{r}_n^{(f)}\}$ is the final configuration of the system, and the Gaussian noise $\boldsymbol{\eta}_n(t)$ satisfies

$$\langle \eta_n^{(\alpha)}(t)\rangle = 0, \quad \langle \eta_n^{(\alpha)}(t)\eta_{n'}^{(\alpha')}(t')\rangle = \frac{2\,\kappa_b T}{\gamma}\delta_{nn'}\delta_{\alpha\alpha'}\delta(t-t')$$

where α labels the Cartesian coordinates x, y, z.

Polymer chain. In the following we specialize eq. (7) to the case of ring polymers that freely evolve under the action of the following inter-monomer potential, U

$$\beta U = \frac{3}{2a^2}\sum_{n=1}^{N}(\mathbf{r}_{n+1}-\mathbf{r}_n)^2 + \frac{K}{2}\sum_{n=1}^{N}(\mathbf{r}_{n+1}-2\mathbf{r}_n+\mathbf{r}_{n-1})^2$$

where β is the inverse temperature and, since the chain is a ring, $\mathbf{r}_N = \mathbf{r}_0$ and $\mathbf{r}_{N+1} = \mathbf{r}_1$.

To model chains with preassigned root-mean-square bond length, b, and persistence length, l_P, the bare parameters a and K must be set as follows:

$$l_P = \sqrt{\frac{Ka^2}{3}} \quad (8)$$

$$b^2 = \frac{1}{N}\langle\sum_{n=1}^{N}(\mathbf{r}_{n+1}-\mathbf{r}_n)^2\rangle \quad (9)$$

$$= \frac{a^2}{N}\sum_{p=0}^{N-1}\left[1 + \frac{2Ka^2}{3}(1-\cos\omega_p)\right]^{-1} \quad (10)$$

where $\omega_p = \frac{2\pi}{N}p$. In the limit $N \to \infty$, we have

$$b^2 = a^2\int_{-\pi}^{+\pi}\frac{d\omega}{2\pi}\frac{1}{1+\frac{2Ka^2}{3}(1-\cos\omega)} \quad (11)$$

and for large K, it can be written as

$$b^2 = a^2\int_{-\pi}^{+\pi}\frac{d\omega}{2\pi}\frac{1}{1+\frac{Ka^2\omega^2}{3}} \ . \quad (12)$$

After some calculations, we obtain

$$l_P = \frac{2}{3}Kb^2 \quad (13)$$

which shows that the persistence length l_P is proportional to the parameter K. In addition, the parameter a is related to the Kuhn length b by

$$a = b\sqrt{2l_P} \ . \quad (14)$$

For the considered polymer case, the Langevin bridge equation of (7) is best expressed in Fourier space:

$$\frac{d\tilde{\boldsymbol{\rho}}_p}{dt} = -\Omega_p\tilde{\boldsymbol{\rho}}_p + \frac{D}{N}\boldsymbol{\nabla}_{\tilde{\rho}_p}\ln Q + \tilde{\boldsymbol{\eta}}_p \ , \quad (15)$$

where

$$\tilde{\boldsymbol{\rho}}_p = \frac{2}{N}\sum_{n=1}^{N}\cos(\omega_p n)\,\mathbf{r}_n \quad (16)$$

$$\Omega_p = (3/a^2)(1-\cos\omega_p) + 2K(1-\cos\omega_p)^2 \quad (17)$$

and $\tilde{\boldsymbol{\eta}}_p$ are the Fourier series of $\boldsymbol{\eta}_n(t)$ and are thus Gaussian white noises, defined by their moments

$$\langle\tilde{\eta}_p(t)\rangle = 0 \quad (18)$$

$$\langle\tilde{\eta}_0^{(\alpha)}(t)\eta_p^{(\alpha')}(t')\rangle = \frac{2D}{N}\delta_{p0}\delta_{\alpha\alpha'}\delta(t-t') \quad (19)$$

$$\langle\tilde{\eta}_p^{(\alpha)}(t)\eta_{p'}^{(\alpha')}(t')\rangle = \frac{D}{N}\delta_{pp'}\delta_{\alpha\alpha'}\delta(t-t') \ . \quad (20)$$

The Green's function $Q(\tilde{\boldsymbol{\rho}}_p, t)$ can be computed exactly by solving the Langevin equation in Fourier space.

The calculation yields

$$Q(\tilde{\boldsymbol{\rho}}_p, t) = \exp\left(-\frac{N}{D}\Omega_p\frac{\left(\tilde{\boldsymbol{\rho}}_p^{(f)} - \tilde{\mathbf{R}}_p(t)\right)^2}{1-e^{-2\Omega_p(t_f-t)}}\ ,\right) \quad (21)$$

so that the bridge equations then become

$$\frac{d\tilde{\boldsymbol{\rho}}_0}{dt} = \frac{1}{\gamma}\tilde{\mathbf{F}}_0(t) + \frac{\tilde{\boldsymbol{\rho}}_0^{(f)} - \tilde{\mathbf{R}}_0(t)}{t_f - t} + \tilde{\boldsymbol{\eta}}_0(t)$$

$$\frac{d\tilde{\boldsymbol{\rho}}_p}{dt} = \frac{1}{\gamma}\tilde{\mathbf{F}}_p(t) - \Omega_p\tilde{\boldsymbol{\rho}}_p(t) + \Omega_p\frac{\tilde{\boldsymbol{\rho}}_p^{(f)} - \tilde{\mathbf{R}}_p(t)}{\sinh\Omega_p(t_f-t)} + \tilde{\boldsymbol{\eta}}_p(t)$$

where $\tilde{\boldsymbol{\rho}}_p^{(f)}$ denotes the final configuration of the chain in

Fourier components,

$$\tilde{\mathbf{R}}_0(t) = \tilde{\boldsymbol{\rho}}_0(t) + \frac{1}{\gamma}\int_t^{t_f} d\tau \tilde{\mathbf{F}}_0(\tau)$$

$$\tilde{\mathbf{R}}_p(t) = \tilde{\boldsymbol{\rho}}_p(t) e^{-\Omega_p(t_f - t)} + \frac{1}{\gamma}\int_t^{t_f} d\tau e^{-\Omega_p(t_f-\tau)}\tilde{\mathbf{F}}_p(\tau)$$

and the forces $\tilde{\mathbf{F}}_p(t)$ are the Fourier series of the forces $\mathbf{F}_n(t)$ defined according to eq.(16). Note that these equations bear some resemblance to the bridge equations for an Ornstein-Uhlenbeck process [6], since the original Langevin equations are linear in both cases.

These equations can be discretized and solved numerically, from an initial configuration $\tilde{\boldsymbol{\rho}}_p^{(0)}$ to a final one $\tilde{\boldsymbol{\rho}}_p^{(f)}$.

[1] Orland, H., *Generating transition paths by langevin bridges*, *J. Chem. Phys.*, 134(2011):174114

[2] Kampen, N.V., *Stochastic Processes in Physics and Chemistry* (North–Holland, Amsterdam, The Netherlands, 1992)

[3] Zwanzig, R., *Nonequilibrium Statistical Mechanics* (Oxford University Press, Oxford, United Kingdom, 2001)

[4] Doob, J., *Conditional brownian motion and the boundary limits of harmonic functions*, *Bull. Soc. Math. France*, 85(1957):431–458

[5] Fitzsimmons, P., Pitman, J. and Yor, M., *Markovian bridges: construction, palm interpretation, and splicing*, in *Seminar on Stochastic Processes, 1992* (Birkhaeuser, Boston, MA, USA, 1992)

[6] Majumdar, S. and Orland, H., *Effective langevin equations for constrained stochastic processes*, *J. Stat. Mech. Theor. Exp.*, 2015(2015)(6):P06039
